# Complete Genome Sequences of Seven Neisseria gonorrhoeae Clinical Isolates from Mucosal and Disseminated Gonococcal Infections

**DOI:** 10.1128/MRA.00734-21

**Published:** 2021-10-28

**Authors:** Freda E.-C. Jen, John M. Atack, Jennifer L. Edwards, Michael P. Jennings

**Affiliations:** a Institute for Glycomics, Griffith University, Brisbane, Queensland, Australia; b The Center for Microbial Pathogenesis, The Abigail Wexner Research Institute at Nationwide Children’s Hospital, Columbus, Ohio, USA; c The Department of Pediatrics, The Ohio State University, Columbus, Ohio, USA; University of Rochester School of Medicine and Dentistry

## Abstract

Neisseria gonorrhoeae is a Gram-negative bacterium that causes the sexually transmitted infection gonorrhea. N. gonorrhoeae has progressively developed resistance to all currently prescribed antibiotics, and no vaccine is available. Here, we report the closed, completed, annotated genome sequences for seven N. gonorrhoeae strains obtained by single-molecule real-time (SMRT) long-read genome sequencing.

## ANNOUNCEMENT

Neisseria gonorrhoeae causes the sexually transmitted disease gonorrhea, which is currently one of the most common bacterial infectious diseases worldwide. Gonorrhea commonly presents as urethritis in men and cervicitis in women. Initially, gonorrhea could be easily treated with penicillin; however, it has since developed resistance to each successive recommended treatment, placing a burden on health care systems and threatening to breach last-line antibiotic treatment (1). No N. gonorrhoeae vaccines are available (2). Whole-genome sequencing of N. gonorrhoeae will provide a useful tool for unraveling the pathogenesis of this important bacterial species. There are many complete N. gonorrhoeae genome assemblies in the NCBI Reference Sequence Database; however, most of these were obtained using short-read Illumina sequencing. One limitation of short read lengths is that many repetitive features such as simple DNA sequence repeats (SSRs) and gene duplications may be lost during automated assembly (3). There are at least 36 translational, phase-variable genes in N. gonorrhoeae (4). In addition, N. gonorrhoeae contains 19 copies of silent, variable *pilS* genes, which can recombine with the pilin expression gene (5). Therefore, long-read sequencing (e.g., single-molecule real-time [SMRT]) is important for obtaining closed, complete genome sequences for N. gonorrhoeae pathogenesis research. Here, we used SMRT sequencing to sequence seven N. gonorrhoeae strains, 1291 (6), MS11 (7), O1G1370 (8, 9), 88G285 (8, 9), O2D156 (8), 98D159 (8, 9), and SK92-679 (10), isolated from mucosal and disseminated gonococcal infections, and report their closed, annotated whole-genome sequences. The improved synteny of these genome sequences will be useful for studying phase-variable and duplicate genes in N. gonorrhoeae.

N. gonorrhoeae strains were grown on GC agar supplemented with 1% IsoVitaleX (BD BBL) at 37°C and 5% CO_2_ overnight and subcultured for 4 h. Cells were harvested from the plates, and genomic DNA was prepared using the GenElute kit (Sigma-Aldrich); PacBio long-read sequencing was carried out at SNPsaurus (Eugene, OR). SMRTbell libraries were prepared using the Express template prep kit 2.0 according to the manufacturer’s protocol (Pacific Biosciences, CA). The samples were pooled into a single multiplexed library and size selected using Sage Sciences’ BluePippin (BP) system according to the manufacturer’s recommendations, with the 0.75% DF marker S1 high-pass 6 kb to 10 kb v3 run protocol and S1 marker. A size selection cutoff of 8,000 (BP start value) was used. The size-selected SMRTbell library for each strain was annealed and bound according to the SMRT Link setup, pooled, and sequenced using Sequel II chemistry v1.0 at SNPsaurus. The raw reads were converted to FASTA format using SAMtools (11). Flye v2.8 (12) was used to assemble and polish the sequenced genomes. The assembly quality was assessed using BUSCO v3 (13). Default parameters were used for all software unless otherwise specified. An average coverage of ∼195-fold was obtained. The assembled sequences were annotated using the Prokaryotic Genome Annotation Pipeline (PGAP) during NCBI GenBank submission of the closed genome sequences (14). Information for each strain/genome/plasmid is summarized in Table 1.

**TABLE 1:** Summary of information for the closed annotated genome and plasmid sequences for seven strains of N. gonorrhoeae

Strain	Type of infection^*a*^	Genome size (bp)	Genome coverage (×)	Total no. of reads (bases)	*N*_50_ (bp)	GC content (%)	No. of genes	No. of CDS^*b*^	GenBank accession no.	SRA accession no.	Plasmid found and sequenced	Plasmid homology:	
												%	With (plasmid name [GenBank accession no.])
1291	MI	2,177,032	275	635,585,034	20,140	52.6	2,276	2,208	CP078119	SRX11344351	Plasmid 1	100	WHO_O plasmid 4 (LT592149.1)
MS11	MI	2,234,079	289	669,009,143	20,168	52.4	2,350	2,279	CP078118	SRX11344352			
O1G1370	MI	2,215,052	167	424,231,185	19,999	52.4	2,327	2,256	CP078115	SRX11344355	Plasmid 1	99	WHO_W plasmid 2 (LT592164.1)
88G285	DGI	2,174,189	127	319,137,608	18,991	52.6	2,254	2,183	CP078116	SRX11344354	Plasmid 1	99	WHO_O plasmid 3 (LT592148.1)
O2D156	DGI	2,165,933	250	571,663,940	20,852	52.6	2,264	2,193	CP078113	SRX11344357			
98D159	DGI	2,172,572	168	403,272,244	19,828	52.6	2,271	2,200	CP078114	SRX11344356	Plasmid 1	99	WHO_M plasmid 4 (LT591907.1)
SK92-679	DGI	2,173,187	91	219,520,879	19,212	52.6	2,265	2,194	CP078117	SRX11344353	Plasmid 1	100	WHO_G plasmid 2 (LT591899.1)

a MI, mucosal infection; DGI, disseminated gonococcal infection.

b CDS, coding DNA sequences.

### Data availability.

The genome sequences and whole-genome sequencing (WGS) reads have been deposited at NCBI. The accession numbers for the closed genome sequences and the raw data are provided in Table 1. The master record for the WGS reads and closed annotated genome sequences can be found at NCBI under BioProject accession number PRJNA743132.
